# Oral Health-Related Quality of Life in Adult Patients with Newly Diagnosed Acute Leukaemia

**DOI:** 10.3290/j.ohpd.a44685

**Published:** 2020-07-04

**Authors:** Gerhard Schmalz, Rilana Busjan, Marit Dietl, Justin Hasenkamp, Lorenz Trümper, Dirk Ziebolz

**Affiliations:** a Doctor and Researcher, Department of Cariology, Endodontology and Periodontology, University of Leipzig, Germany. Data analysis and interpretation; wrote the manuscript.; b Doctor and Researcher, Department of Cariology, Endodontology and Periodontology, University of Leipzig, Germany. Performed data curation and revised the manuscript.; c Department of Cariology, Endodontology and Periodontology, University of Leipzig, Germany. Performed analysis and revised the manuscript.; d Doctor and Researcher, Clinic for Hematology and Medical Oncology, University of Goettingen, Germany. Data curation and interpretation; revised the manuscript.; e Professor and Head, Clinic for Hematology and Medical Oncology, University of Goettingen, Germany. Supervised the study and revised the manuscript.; f Professor and Researcher, Clinic for Hematology and Medical Oncology, University of Goettingen, Germany. Head of the study, participated in data analysis and interpretation and revised the manuscript.

**Keywords:** acute leukaemia, oral health-related quality of life, oral initial symptoms, oral health

## Abstract

**Purpose:**

Knowledge about oral health-related quality of life (OHRQoL) in adult patients with leukaemia is still limited. Accordingly, aim of this cross-sectional study was to assess OHRQoL and its associations to different parameters in adult patients with newly diagnosed acute leukaemia.

**Materials and Methods:**

Participants with first diagnosis of acute leukaemia were consecutively recruited in the Clinic of Hematology and Oncology of the University Medical Center Goettingen. OHRQoL was assessed using the German short form of oral health impact profile (OHIP-G14). Presence of oral initial symptoms, dental health (decayed- [D-T], missing- [M-T] and filled-teeth index [DMF-T]), dental behaviour and periodontal disease severity were assessed. For comparison, a healthy control group (HC) was recruited.

**Results:**

Thirty-nine patients with leukaemia and 38 HC were included. In the leukaemia group, a statistically significant and clinically relevant higher OHIP-sum score compared to HC was found (6.13 [3; 0–7] vs 0.87 [0; 0–2], p <0.01). The different subaspects of OHRQoL (patterns) ‘oral function’ and ‘orofacial appearance’ were statistically significantly worse in the leukaemia group (p <0.01). Time since diagnosis showed a clinically relevant association to the pattern ‘psychosocial impact’ (p = 0.06). Patients with oral initial symptoms had a statistically significantly worse OHIP-sum score (p <0.04, V = 0.775). DMF-T (p = 0.03, r = 0.242) and M-T (p = 0.03, r = 0.252) showed an association to OHIP sum score. Moreover, D-T (p = 0.03, r = 0.253) and M-T (p = 0.01, r = 0.296) were associated to orofacial appearance. Additionally, M-T showed an association to pattern ‘oral function’ (p = 0.01, r = 0.277).

**Conclusion:**

Patients with newly diagnosed acute leukaemia show a reduced OHRQoL. This might be particularly caused by oral health situation, especially oral initial symptoms as well as missing teeth.

Acute leukaemia encloses a heterogeneous group of cancers affecting the blood, including the two main types acute myeloid (AML) as well as acute lymphoblastic leukaemia (ALL).^3,17^ These diseases lead to rapid inhibition of haematopoiesis and lead, if untreated, to a fatal outcome.^3^ Severe symptoms resulting from the pancytopenia are fatigue, pallor, weakness, susceptibility to infections and increased bleeding tendency.^3^ In this context, it is not surprising that the quality of life is often affected by the severe disease.^13^ Thereby, fatigue, depression and anxiety are very present in this patients group at the time of diagnosis, during and after therapy.^13^

Furthermore, oral health conditions are of relevance in the group of patients suffering from acute leukaemia. Different oral manifestations of the disease are described, including hyperplastic gingiva, bleeding and petechiae, mucosal pallor and bacterial, viral and fungal infections.^2,14,30^ As these symptoms are known to be early manifestations of the disease, dentists are involved in approximately 25% of diagnosis of acute leukaemia cases.^30^ Although data about the oral health situation of this patients group are rare, an insufficient oral health is stated in the available studies.^2,14,30^ However, most available studies are focused on patients during therapy. Considering the fact that insufficient oral health might be associated with a higher risk of systemic infections and complications of patients with acute leukaemia,^1,5^ the current situation shows urgent need for improvement. Patients with newly diagnosed acute leukaemia are of particular importance, because early dental treatment, including the elimination of potential infectious dental foci before chemotherapy or stem cell transplantation might reduce fatal consequences.^5,6^

Nevertheless, it is up until now unclear in what way patients with acute leukaemia are affected by their oral health situation, or if the high psychological burden of their diagnosis makes oral health a minor matter. In this context, assessment of oral health-related quality of life (OHRQoL), which is part of the general health-related quality of life,^22^ might be a promising approach to gain insight into this issue. It has been shown in several studies that patients with severe diseases or conditions like organ transplantation or rheumatic diseases suffering from a reduced OHRQoL, independently of their insufficient oral conditions.^18,26,27^ This might lead to a reduced dental behaviour and delay or even absence of dental consultations and rehabilitation, what could be similar in patients with acute leukaemia.

Therefore, the current study aimed in the investigation of OHRQoL in a group of patients with newly diagnosed, untreated acute leukaemia. It was hypothesised that these patients show reduced OHRQoL, independently of their oral conditions.

## Materials and Methods

### Study Design

The current study was performed as a cross-sectional study and included adult patients with newly diagnosed acute leukaemia. The oral health situation of these patients has already been assessed in a previous study by this working group.^2^ The study has been reviewed and approved by the Ethics Committee of the University Medical Center in Goettingen, Germany (No. 30/1/14). All patients were informed verbally and in writing and gave their written informed consent for participation.

### Patients

Between January and April 2015, patients with newly diagnosed acute leukaemia were consecutively recruited during their appearance and hospitalisation in the Clinic of Hematology and Oncology of the University Medical Center Goettingen to receive induction chemotherapy. All examinations, including assessment of OHRQoL were performed before the onset of any therapeutic intervention. Both patients with ALL and AML were recruited and investigated. The following exclusion criteria were formulated: age under 18 years (patients with a minimum age of 18 years were defined as ‘adults’), general condition that prohibited oral examination (eg, severe infection), pregnancy, autoimmune diseases as well as severe immunosuppression (severe neutropenia or thrombocytopenia).

Furthermore, a healthy control group (HC) has been recruited within the same time period and consisted of patients who were newly admitted to the Department of Preventive Dentistry, Periodontology and Cariology of the University Medical Center Goettingen. The HC group was well matched regarding age (per year) and gender (male or female). Smoking status was assessed for both groups, whereby patients who currently smoked were defined as ‘smoker’, patients who stopped smoking before less than 5 years were defined as ‘former smoker’ and patients who never smoked or were smokers more than 5 years ago were seen as ‘non-smoker’.

### Oral Examination

The oral examination was performed as described previously.^2^ In brief, the decayed- (D-T), missing- (M-T) and filled-teeth index (DMF-T) was assessed according to the WHO.^29^ Moreover, the presence of mucosal changes in the oral cavity was recorded. In this context, patients were asked whether there were oral initial symptoms. The occurrence of hyperplasia, petechiae, bleeding, necrotic tissue and bacterial, viral or mycotic infections at gums, lips, cheeks, palate, floor of mouth and pharynx were recorded as oral initial symptoms. The assessment of periodontal conditions was executed as a six-point-measurement per tooth, using a millimetre-scaled periodontal probe (PCP 15, HU-Friedy, Chicago, IL, USA). In accordance to Page and Eke,^20^ periodontal conditions were categorised into (1) no/mild periodontitis, (2) moderate periodontitis, (3) severe periodontitis.

### Dental Behaviour and OHRQoL

Patients were asked about their dental behaviour, ie, regularity of dental consultations (if patients regularly contact the dentist or not), a dental treatment before diagnosis (any invasive dental treatment within the past year before diagnosis) and the presence of a dental rehabilitation (invasive dental treatment to eliminate potential infectious dental foci) since diagnosis using a questionnaire. For assessment of OHRQoL, the German short form of the oral health impact profile (OHIP-G14) was applied.^8,28^ The OHIP G14 is a standardised and validated questionnaire, indicating 14 functional and psychosocial impacts which patients experienced within the previous month as a result of problems with their teeth, mouth or dentures. Answers are graduated as follows: 0 = ‘never’, 1 = ‘hardly ever’, 2 = ‘occasionally’, 3 = ‘fairly often’ and 4 = ‘very often’. According to John et al (2016), the different questions of the OHIP-G14 questionnaire were categorised into the patterns ‘oral function’, ‘psychosocial impact’, ‘oral pain’ and ‘orofacial appearance’.^10,11^ According to Reissmann et al (2008), differences in OHIP-G14 values of on average 2 or more points between groups were interpreted as clinically relevant.^21^

### Statistical Analysis

Statistical analysis was carried out using the statistical program SPSS, Version 22.0 (SPSS, IBM, New York, NY, USA). The investigated parameters were tested for their normal distribution using the Kolmogorov–Smirnov test, whereby age and F-T were found to be normal distributed (p >0.05). For comparison of two non-normal distributed, independent samples, the Mann-Whitney U test was applied. Categorical samples were analysed with Chi-square test. In case of statistical significance, correlation of variables was performed with Cramer’s V or Spearman’s Rho test, respectively. The statistical significance level was determined as p <0.05.

## Results

### Patients

In total, 39 patients (19 male, 20 female; 26 AML, 13 ALL) diagnosed with acute leukaemia were included. Furthermore, a HC group consisting of 38 participants was examined. Between the Leukaemia and HC groups, comparable age and gender was recorded (p >0.05), while smoking habits were statistically significantly different between the groups (p = 0.02; Table 1).

**Table 1:** Patients’ characteristics in leukaemia group and healthy control

		Overall (n = 77)	Leukaemia (n = 39)	HC (n = 38)	P value
**Age [mean value ± standard deviation (median)]**		55.6 ± 16.21 (56, 5)	55.59 ± 16.79 (56)	55.62 ± 15.84 (57)	0.99*
**Gender n (%)**	**female**	39	20 (51.3)	19 (50)	0.91**
	**male**	38	19 (48.7)	19 (50)	
**Non-smoker**		47	19	28	0.02**
**Former smoker**		13	11	2	
**Smoker**		17	9	8	

*t test, **Chi-square test.

### OHRQoL Between Leukaemia and HC

A comparison of OHIP-findings between the two groups is presented in Table 2. In the leukaemia group, a statistically significant and clinically relevant higher OHIP-sum score compared to HC was found (6.13 [3; 0–7] vs 0.87 [0; 0–2], p <0.01). The different patterns, ‘oral function’ (2.13 [0; 0–3] vs 0.05 [0; 0–0]; p <0.01) and ‘orofacial appearance’ (1.03 [0; 0–2] vs 0.05 [0; 0–0]; p <0.01) were statistically significantly worse in leukaemia group. Thereby, only the difference in the pattern ‘oral function’ was clinically relevant, according to Reissmann et al (2008).^21^ The other two patterns ‘psychosocial impact’ and ‘oral pain’ were neither statistically significant nor clinically relevant different between the groups (p >0.05).

**Table 2:** OHIP scores and different patterns between leukaemia and healthy control group. Values are given as mean (median; 25th–75th percentile)

	Leukaemia	HC	P value
**OHIP**	6.13 (3; 0–7)	0.87 (0; 0–2)	<0.01
**Oral function** Trouble pronouncing Taste worsened Interrupting meals Uncomfortable to eat Diet unsatisfactory	2.13 (0; 0–3) 0.21 (0; 0–0) 0.49 (0; 0–0) 0.28 (0; 0–0) 0.77 (0; 0–1) 0.38 (0; 0–0	0.05 (0; 0–0) 0 (0; 0–0) 0.03 (0; 0–0) 0 (0; 0–0) 0 (0; 0–0) 0.03 (0; 0–0)	<0.01 0.04 0.04 0.08 0.01 0.0
**Psychosocial impact** Life less satisfying Difficult to relax Feeling of tension Short tempered Difficulty performing jobs Unable to function Embarrassed	2.18 (0; 0–2) 0.31 (0; 0–0) 0.28 (0; 0–0) 0.46 (0; 0–0) 0.23 (0; 0–0) 0.33 (0; 0–0) 0.26 (0; 0–0) 0.31 (0; 0–0)	0.46 (0; 0–1) 0.03 (0; 0–0) 0.26 (0; 0–0) 0.15 (0; 0–0) 0 (0; 0–0) 0 (0; 0–0) 0 (0; 0–0) 0.03 (0; 0–0)	0.39 0.04 0.77 0.60 0.02 0.02 0.04 0.08
**Orofacial pain**			
Oral pain	0.79 (0; 0–2)	0.31 (0; 0–0)	0.06
**Orofacial appearance**			
Sense of uncertainty	1.03 (0; 0–2)	0.05 (0; 0–0)	<0.01

Main categories are highlighted in bold, statistical significance level p <0.05 (Mann-Witney U test).

### Correlation of OHRQoL to Different Parameters

Association of OHRQoL to different parameters are presented in Table 3. The form of leukaemia was not associated to OHIP scores (AML: 6.92 [3; 0–7.25] vs ALL: 4.54 [2; 0–6.5]; p = 0.90). Regarding time since diagnosis (0–2 vs >2 days), increased time since diagnosis showed a clinically relevant association to pattern ‘psychosocial impact’, which has been confirmed by a statistical trend (1.4 [0; 0–1.25] vs 4.78 [1; 0–11]; p = 0.06). Patients with oral initial symptoms had a statistically significantly worse OHIP-sum score (16.56 [8; –4–30.5] vs 3 [1.5; 0–4]; p <0.04, V = 0.775), which was also detectable in the patterns ‘oral function’ (6.56 [4; 0.5–13] vs 0.8 [0; 0–0.25]; p = 0.03, V = 0.667), ‘oral pain’ (2.11 [2; 0–4] vs 0.4 [0; 0–0.25]; p <0.01, V = 0.632) and ‘orofacial appearance’ (2.33 [3; 0–4] vs 0.63 [0; 0–1]; p = 0.04, V = 0.495). Patients who consulted the dentist since the leukaemia diagnosis showed better OHIP-sum score (3.8 [3; 2.5–5.5] vs 6.47 [1.5; 0–8]; p = 0.05), but worse score in pattern ‘oral pain’ (2 [1; 1–3] vs 0.62 [0; 0–1]; p = 0.02, V = 0.513). Regarding oral health parameters, the DMF-T (p = 0.03, r = 0.242) and M-T (p = 0.03, r = 0.252) showed an association to OHIP sum score. Moreover, D-T (p = 0.03, r = 0.253) and M-T (p = 0.01, r = 0.296) were associated to orofacial appearance. Additionally, M-T showed an association to pattern ‘oral function’ (p = 0.01, r = 0.277).

**Table 3:** Association of OHIP scores to disease specific parameters, dental behaviour as well as dental health parameters in leukaemia group. Values are given as mean (median; 25th–75th percentile)

Variable	OHIP-Sum	Oral function	Psychosocial impact	Oral pain	Oral appearance
**Form of leukaemia**					
**AML (n = 26)** **ALL (n = 13)**	6.92 (3; 0–7.25) 4.54 (2; 0–6.5)	2.42 (0; 0–4) 1.54 (0; 0–3)	2.35 (0; 0–2) 1.85 (0; 0–2)	1.12 (0; 0–2) 0.15 (0; 0–0)	1.04 (0; 0–2.25) 1 (0; 0–2)
	p = 0.90	p = 0.39	p = 0.66	p = 0.06	p = 0.778
**Time since diagnosis**					
**0–2 days (n = 30) >2 days (n = 9)**	4.97 (2.5; 0–5.25) 10 (3; 0–17.5)	1.8 (0; 0–2.25) 3.22 (0; 0–7)	1.4 (0; 0–1.25) 4.78 (1; 0–11)	0.73 (0; 0–1.25) 1 (0; 0–2)	1.03 (0; 0–2) 1 (0; 0–2.5)
	p = 0.15	p = 0.09	p = 0.06	p = 0.86	p = 0.56
**Oral initial symptoms**					
**yes (n = 9)** **no (n = 30)**	16.56 (8; 4–30.5) 3 (1.5; 0–4)	6.56 (4; 0.5–13) 0.8 (0; 0–0.25)	5.56 (0; 0–11) 1.17 (0; 0–1.25)	2.11 (2; 0–4) 0.4 (0; 0–0.25)	2.33 (3; 0–4) 0.63 (0; 0–1)
	**p = 0.04** Cramer-V: 0.775	**p = 0.03** Cramer-V: 0.667	p = 0.21	**p <0.01** Cramer-V: 0.632	**p = 0.04** Cramer-V: 0.495
**Dental behaviour**					
**Regular dental treatment**					
**yes (n = 22)** **no (n = 17)**	7.77 (3; 0–7.25) 4 (1; 0–7) p = 0.24	2.59 (0; 0–3) 1.53 (0; 0–4) p = 0.227	3.05 (0; 0–2.25) 1.06 (0; 0–2) p = 0.40	1 (0; 0–2) 0.53 (0; 0–1) p = 0.07	1.14 (0; 0–2.25) 0.88 (0; 0–1.5) p = 0.66
**Consultation of dentist since diagnosis?**					
**yes (n = 5)** **no (n = 32)**	3.8 (3; 2.5–5.5) 6.47 (1.5; 0–8) p = 0.05	0.2 (0; 0–0.5) 2.41 (0; 0–4) p = 0.81	0.6 (0; 0–1.5) 2.41 (0; 0–2.25) p = 0.68	2 (2; 1–3) 0.62 (0; 0–1) **p = 0.02** Cramer-V: 0.513	1 (0; 0–2.5) 1.03 (0; 0–2) p = 0.38
**Dental treatment before diagnosis?**					
**yes (n = 13)** **no (n = 26)**	7.46 (3; 0–11.5) 5.46 (2; 0–5.25) p = 0.57	2.08 (0; 0–3.5) 2.15 (0; 0–3.25) p = 0.82	3.15 (0; 0–4) 1.69 (0; 0–2) p = 0.27	1.15 (0; 0–2) 0.62 (0; 0–1) p = 0.63	1.08 (0; 0–2.5) 1 (0; 0–2) p = 0.96
**Dental rehabilitation since diagnosis?**					
**yes (n = 3)** **no (n = 36)**	6 (3; 0) 6.14 (2.5; 0–6.75) p = 0.74	2.33 (0; 0) 2.11 (0; 0–3) p = 0.64	2 (0; 0) 2.19 (0; 0–2) p = 0.07	0.67 (0; 0) 0.81 (0; 0–1.75) p = 0.738	1 (0; 0) 1.03 (0; 0–2) p = 0.20
**Oral health**					
**DMF-T** **D-T** **M-T** **Periodontitis** **no/mild (n = 6)** **moderate (n = 16)** **severe (n = 12)**	**p** **= 0.03** r = 0.242 p = 0.36 **p = 0.03** r = 0.252 3.59 (0; 0–2.5) 3.93 (1; 0–3.75) 2.62 (1; 0–4) p = 0.52	p = 0.50 p = 0.53 **p = 0.01** r = 0.277 1.12 (0;0–0) 1.15 (0; 0–0) 0.95 (0; 0–0.5) p = 0.37	p = 0.28 p = 0.24 p = 0.41 1.76 (0; 0–0.5) 1.48 (0; 0–1) 0.67 (0; 0–1.5) p = 0.64	p = 0.38 p = 0.67 p = 0.99 0.35 (0; 0–0) 0.75 (0; 0–2) 0.33 (0; 0–0) p = 0.579	p = 0.06 **p = 0.03** r = 0.253 **p = 0.01** r = 0.296 0.35 (0; 0–0) 0.55 (0; 0–0) 0.67 (0; 0–0) p = 0.672

AML, acute myeloid leukaemia, ALL, acute lymphoblastic leukaemia; DMF-T, number of decayed (D-T), missing (M-T) and filled-teeth. Differences in D-T, M-T and DMF-T were investigated based on the median (above vs below median). Statistically significant results are highlighted in bold (p <0.05).

## Discussion

In the current cross-sectional study, patients with newly diagnosed acute leukaemia showed worse OHIP-sum, ‘oral function’ and ‘orofacial appearance’ score compared to HC. Within the leukaemia group, time since diagnosis trended to be associated to psychosocial impact. The presence of oral initial symptoms resulted in worse OHIP-sum, ‘oral function’, ‘oral pain’ and ‘orofacial appearance’ scores. Moreover, particularly higher M-T showed correlations to OHIP-sum, ‘oral function’ and ‘orofacial appearance’ score.

It must be mentioned that this is the first assessment of OHRQoL of patients with newly diagnosed acute leukaemia. Consequently, there is no comparable literature available for this patient group. In total, OHIP values for leukaemia group was 6.13 (3; 0–7) and for HC a sum score of 0.87 (0; 0–2) was found. Considering reference values for healthy general population defined by John et al (2004), a range between 0 and 4 for healthy fully or partially dentate individuals was defined as reference.^9^ While the HC lies within this range, values for leukaemia group are higher. Accordingly, the OHRQoL of adult patients with newly diagnosed acute leukaemia can be stated as reduced. The scale of the reduction of OHRQoL is comparable to German patients suffering from rheumatoid arthritis (OHIP: 7.3) and ankylosing spondylitis (OHIP: 6.2).^18,26^ Other medically compromised patients undergoing haemodialysis or after solid organ transplantation showed lower OHIP values between 1.7 and 4.1, which lie within the predefined reference range.^9,24,25^ In contrast, generally healthy patients with oral diseases as periodontitis or temporomandibular disorders show reduction that is more pronounced in OHRQoL.^4,16,23,24^ Accordingly, the OHRQoL impairment of leukaemia patients seems to be well in line and similar as for special groups of seriously medically compromised patients. However, while in the other medically compromised patient groups no association between OHIP scores and oral health parameters were found, leukaemia patients showed in contrast deficiency of DMF-T as well as the presence of oral initial symptoms. Therefore, the pattern oral function showed the most relevant impairment. Different approaches might explain this situation. Patients with acute leukaemia often suffer from oral initial symptoms like hyperplastic gingiva, bleeding and petechiae, mucosal pallor and bacterial, viral and fungal infections.^2,14,30^ It has been described, that oral mucosal diseases affect general and OHRQoL.^15,19^ Accordingly, the OHIP-score is strongly associated to the presence of oral symptoms of leukaemia. Thereby, OHIP-sum score, as well as the patterns oral function and oral pain showed a noteworthy correlation to these oral initial symptoms, while for orofacial appearance a weaker correlation was detected. In this context it must be considered that the OHIP-score of patients without oral initial symptoms was found to be within the reference values.^9^ Accordingly, the presence of oral initial symptoms might be seen as the major reason for impairment in OHRQoL in adults with untreated acute leukaemia. Another aspect is the fact that the general health-related quality of life of patients with acute leukaemia is reduced.^12,13^ This might also affect OHRQoL as a part of the general quality of life. However, most available investigations consider patients undergoing therapy or survivors after treatment. In contrast, the current study examined untreated and newly diagnosed individuals. Thereby, an interesting issue could be the association between psychosocial impact and time since diagnosis. Based on these findings, the psychosocial impairment might appear not immediately after diagnosis. This could explain the difference to other chronically medically compromised patients, which have their disease for many years at the time point of examination. However, the real impact of leukaemia diagnosis on psychosocial aspects of OHRQoL remains unclear and would be just speculative.

A further conspicuous finding was that patients who visited a dentist since the time of diagnosis showed more impairment of the pattern oral pain. It is therefore conceivable, that patients visited their dentist because of oral initial symptoms or other dental problems that caused the pain. Another potential explanation is that patients that avoided dental consultations for prolonged time period visited the dentist because of the allocation by their haematologist. However, this aspect cannot be completely clarified by the current study´s results. The fact that especially the M-T was associated to OHRQoL is not surprising, as this finding confirms the available literature.^7^ However, the correlation of dental findings with OHIP values is moderate in the current study.

This is the first study investigating the OHRQoL in adult patients with newly diagnosed acute leukaemia. The question is of clinical relevance, because the knowledge about quality of life issues is an important factor to improve dental care in this patient group with a special need for sufficient dental rehabilitation. Considering that acute leukaemia is a rare and very severe disease, the inclusion of 39 patients is a further strength of the study. Furthermore, the usage of the OHIP as a validated instrument and the investigation of the different patterns strengthen the findings. However, the patient number might be too low to draw more advanced conclusions, especially in the subgroup analysis regarding oral health and dental behaviour. Moreover, the relevance and reasons for the psychosocial impairment remains unclear, because no information about the general psychological status as well as general quality of life was assessed. However, this might be helpful to gain more insight into the patients’ general psychological impairments.^15^ Additionally, the fact that no sociodemographic parameters, such as education, income, and so on, were assessed is a further limitation of the current study. The design as a cross-sectional study does not allow drawing conclusions regarding the influence of leukaemia on OHRQoL; however, a prospective approach starting before leukaemia diagnosis seems impossible.

## Conclusion

Within the limitations of the small sample size and the study design, adult patients with newly diagnosed acute leukaemia show a reduced OHRQoL. This might be mainly caused by the presence of oral initial symptoms. Furthermore, the number of missing teeth is associated to reduced OHRQoL in these patients.
